# Evidence for an ancient *BRCA*1 pathogenic variant in inherited breast cancer patients from Senegal

**DOI:** 10.1038/s41525-020-0114-7

**Published:** 2020-01-31

**Authors:** Rokhaya Ndiaye, Jean Pascal Demba Diop, Violaine Bourdon-Huguenin, Ahmadou Dem, Doudou Diouf, Mamadou Moustapha Dieng, Pape Saloum Diop, Serigne Modou Kane Gueye, Seydi Abdoul Ba, Yacouba Dia, Sidy Ka, Babacar Mbengue, Alassane Thiam, Maguette Sylla Niang, Papa Madieye Gueye, Oumar Faye, Philomene Lopez Sall, Aynina Cisse, Papa Amadou Diop, Hagay Sobol, Alioune Dieye

**Affiliations:** 1 Laboratory of Cytology, Cytogenetics and Reproductive Biology, Aristide Le Dantec Hospital, Dakar, Senegal; 2 Faculty of Medicine, Pharmacy and Odontology, University Cheikh Anta Diop, Dakar, Senegal; 3 African Center of Excellence for Mother and Child Health (CEA-SAMEF), Dakar, Senegal; 4 Laboratory of Molecular Oncogenetics, Paoli-Calmette Institute, Marseille, France; 5 Joliot Curie Institute, Aristide Le Dantec Hospital, Dakar, Senegal; 6 Pasteur Institute of Dakar, Dakar, Senegal

**Keywords:** Breast cancer, Cancer genetics

## Abstract

*BRCA1* and *BRCA2* are the most incriminated genes in inherited breast/ovarian cancers. Several pathogenic variants of these genes conferring genetic predisposition have been described in different populations but rarely in sub-Saharan Africa. The objectives of this study were to identify pathogenic variants of the *BRCA* genes involved in hereditary breast cancer in Senegal and to search for a founder effect. We recruited after free informed consent, 27 unrelated index cases diagnosed with breast cancer and each having a family history. Mutation screening of the genes identified a duplication of ten nucleotides c.815_824dupAGCCATGTGG, (p.Thr276Alafs) (NM_007294.3) located in exon 11 of *BRCA1* gene, in 15 index cases (allelic frequency 27.7%). The pathogenic variant has been previously reported in African Americans as a founder mutation of West African origin. Haplotypes analysis of seven microsatellites surrounding the *BRCA1* gene highlights a shared haplotype encompassing ~400 kb between D17S855 and D17S1325. This haplotype was not detected in none of 15 healthy controls. Estimation of the age of the pathogenic variant suggested that it occurred ~1400 years ago. Our study identified a founder pathogenic variant of *BRCA1* predisposing to breast cancer and enabled the establishment of an affordable genetic test as a mean of prevention for Senegalese women at risk.

## Introduction

With a rapidly evolving incidence, breast cancer is currently the first female cancer in sub-Saharan Africa followed by cervical cancer.^1^ Recent reviews have reported that most breast cancers in Sub-Saharan Africa are triple negative, prognostic stage III tumours, with average age at diagnosis at late 40 s, resulting in a high-mortality rate.^2,3^ Overall, 5–10% of breast cancers are inherited and could be associated with ovarian cancers. The risk is linked to two high-penetrance susceptibility genes: *BRCA*1 (17q21) and *BRCA2* (13q12). Both are tumour suppressor genes involved in double strand break DNA repair. Women who have inherited mutations in *BRCA1* or *BRCA2* are at higher risk of developing breast and/or ovarian cancers.^4^ Risk increased with the number of affected women within the family, early age at diagnosis and the degree of relationship with other affected women.^5–7^ The cumulative risk of breast cancer by age 80 years was estimated to 72% for *BRCA1* carriers and 69% for *BRCA2* carriers. For ovarian cancer, cumulative risk at age 80 years was estimated to 44% for *BRCA1* carriers and 17% for *BRCA2* carriers.^5–7^

Many studies in different populations have identified pathogenic variants that have been stored in *BRCA* Consortia databases (CIMBA http://cimba.ccge.medschl.cam.ac.uk/, UMD http://www.umd.be/BRCA1/, BIC http://research.nhgri.nih.gov/bic/, ENIGMA https://enigmaconsortium.org/, BRIDGE https://bridges-research.eu/). Some variants are at very high frequencies in specific ethnic groups suggesting their founder effect. In Ashkenazi Jewish women, *BRCA1* c.66_67delAG (p.Glu23Valfs) and c.5266dupC (p.Gln1756Profs) founder mutations conferred a life time risk to breast/ovarian cancer, ten times higher compared with general population.^8,9^ In Africa few studies have reported specific founder mutations of *BRCA1*: c.5335delC (p.Gln1779Asnfs) identified in Egypt^10^; c.5309G>T (p.Gly1770Val) identified in five unrelated families from Morocco^11^; c.303T>G (p.Tyr101Ter) reported in Yoruban population^12^ from Nigeria and c.2641G>T (p.Glu881Ter) in Afrikaner population from South Africa.^13,14^ In addition to these founder mutations, other mutations with African origin have been described in African Americans in the US, in African diaspora and in Hispanics from Peru, Mexico and the Bahamas: c.815_824dupAGCCATGTGG (p.Thr276Alafs**)**; c.1713_1717delAGAAT (p.Glu572Thrfs), and c.5173_5176delGAAA (p.Arg1726Lysfs).^15–19^ Among these mutations c.815_824dup10 is of particular interest. It has been reported as originated from West Africa during the slavery period.^20^ In the CIMBA database this mutation has been identified in 65 people from France, Spain and the US. They are mostly labelled as being of African or Hispanic descent. Haplotypes analyses have shown that the shared *BRCA*1 region flanking c.815_824dup10 is shorter than those flanking European founder mutations.^20–22^ Therefore c.815_824dup10 African mutation may probably be older than European mutations. In West Africa very few studies have screened for *BRCA1* founder mutations.^12,13^ Here we report the highest occurrence of c.815_824dup10 of *BRCA*1 gene in Senegalese patients with inherited breast cancer and confirm its West African origin.

## Results

### Age at diagnosis and tumor characteristics

Mean age at breast cancer diagnosis was 39.5 years (range from 21 to 67 years). Overall, 88.8% of recruited patients were diagnosed before age 50. Of patients with information on tumor stage (94.1%), most were diagnosed at advance stage (stage II = 58.9% or stage III = 35.2%). Tumour hormone receptors and HER2 status showed that 42.8% had triple negative breast cancer followed by HER2 enriched tumors, 28.5 % (Table 1).

**Table 1:** Age at diagnosis and tumor characteristics of studied population.

	Number	Percentage %
Age at diagnosis		
20–35	11	40,7
36–50	13	48,1
>50	3	11,1
SBR stage		
I	1	5,9
II	10	58,9
III	6	35,2
Hormone receptors and HER2 status		
TNBC	6	42,8
HER2 enriched	4	28,5
Other phenotypes	4	28,7
ND	13	48,1

### Identification of a recurrent pathogenic variant of BRCA1 gene

**Fig. 1:**
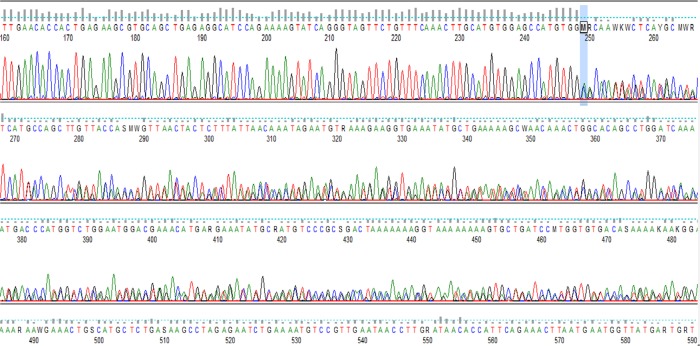
Chromatographic sequence of the *BRCA*1gene exon 11 surrounding the identified pathogenic variant c.815_824dup.

**Fig. 2:**
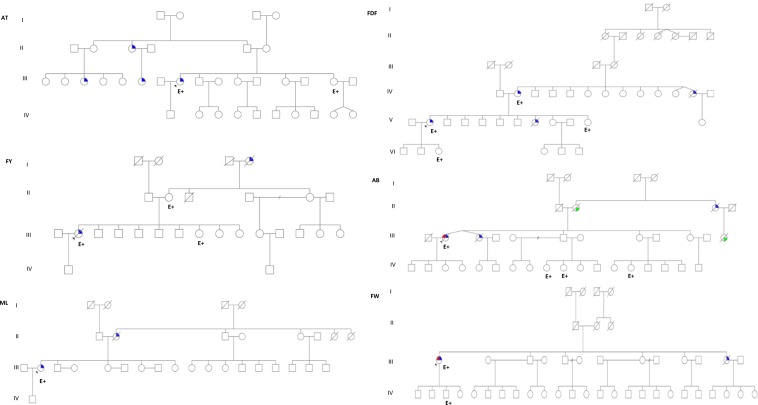
Pedigrees of six families carrying the *BRCA1* pathogenic variant c.815_824dup10 identified by Sanger sequencing. Blue color indicates individuals diagnosed with breast cancer. P: index case, E+: Individual with pathogenic variant, E−: Individual without pathogenic variant.

**Fig. 3:**
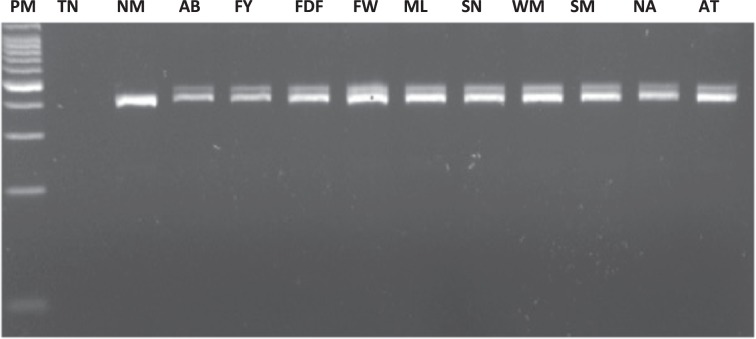
4% agarose gel electrophoresis of PCR products for *BRCA1* pathogenic variant c.815_824dup10 genotyping. PM molecular weight marker, TN DNA negative control, NM non-carrier index case, AB-FY-FGF-FW-ML-SN-WM-SM-NAG-AT: index cases carrying the mutation (AB-FY-FGF-FW-ML-AT belong to the first group of 15 index cases recruited and SN-WM-SM-NAG belong to the second group of 12 recruited index cases). This gel derived from a single experiment.

Mutation screening identified a recurrent pathogenic variant at heterozygous state of the *BRCA*1 gene in 15 probands out of 27 recruited. This is a duplication of ten nucleotides (c.815_824dupAGCCATGTGG, p.Thr276Afs) located in exon 11 of *BRCA*1 according to the HGVS nomenclature (Fig. 1). This pathogenic variant leads to a frameshift and a spurious stop codon 14 amino acids further down. It was detected in six index cases of a first group of 15 index cases by mutation screening of all coding exons of *BRCA1* gene, and later in nine additional index cases from a second group of 12 patients by PCR genotyping (Supplementary Fig. 1). The allelic frequency was then estimated at 27.7% in hereditary breast cancer cases. The pathogenic variant was also detected by genotyping in a control population of sporadic breast cancer cases and healthy controls free from any cancer (allelic frequency estimated at 5% and 0.55%, respectively), and in ten healthy relatives from selected studied families (Table 2, Fig. 2). The PCR genotyping method is now available for first routine screening of the recurrent pathogenic variant in women at risk in our laboratory (Fig. 3).

**Table 2:** c.815_824dup10 pathogenic variant status in the study population.

Mutation status	Families				Sporadic cases		Healthy controls	
	Nb cases	Nb cases	Allelic frequency	Healthy relatives Nb cases	Nb cases	Allelic frequency	Nb cases	Allelic frequency
c.815_824dup10+	6	9	27.7%	10	8	5%	1	0.55%
c.815_824dup10−	9	3		13	72		89	
Total	15	12		23	80		90

Any other pathogenic variant of the *BRCA*1 gene was identified in the remaining nine index cases of the first group while one of them had a novel *BRCA*2 pathogenic variant.^23^

### An ancient founder haplotype linked to the recurrent pathogenic variant

As the pathogenic variant is recurrent in our study population, we searched for its founding effect by haplotype analysis. We genotyped seven microsatellite markers flanking the *BRCA1* gene and distributed in 2.15 Mb in ten index cases with the pathogenic variant, and 15 unrelated healthy controls. Haplotype analysis showed that specific alleles sizes 144, 156, and 173 bp of three microsatellite markers D17S855, D17S1323, and D17S1325, respectively segregated together in the index cases, and constituted a common haplotype of ~400 kb. This haplotype is not found in any of the healthy controls studied. This common haplotype suggested a founder effect of the pathogenic variant in the study population (Table 3).

**Table 3:** Haplotypes of ten probands with the *BRCA1* c.815_824dup10 mutation and 15 healthy controls.

	Patient	D17S1793		D17S1320		D17S855		D17S1323		D17S1325		D17S951		D17S1183	
Index cases	AB1	193	195	186	188	**144**	146	**156**	**156**	171	**173**	170	178	129	146
	AT	193	195	188	190	**144**	144	**156**	158	157	**173**	172	174	135	146
	FDF	193	193	195	195	**144**	152	150	**156**	**173**	175	168	172	120	120
	FW	193	195	186	186	**144**	152	154	**156**	171	**173**	170	172	146	150
	FY1	193	193	224	224	**144**	152	**156**	158	**173**	175	172	174	129	148
	ML1	193	193	213	224	**144**	148	150	**156**	**173**	173	172	172	124	139
	NA	195	195	195	195	**144**	150	154	**156**	175	179	170	178	124	124
	SM	193	193	190	190	**144**	144	**156**	162	169	**173**	168	170	124	146
	SN	193	193	192	192	**144**	148	**156**	156	**173**	175	174	176	124	124
	WM	193	197	203	224	140	**144**	**156**	158	167	171	170	172	120	129
Healthy controls	T33	192	200	193	196	152	152	150	154	171	177	168	172	121	121
	T37	193	195	187	193	152	152	152	154	169	169	170	174	121	121
	T38	192	193	191	193	152	155	152	154	171	**173**	168	172	121	129
	T141	192	195	185	189	**144**	154	**156**	159	157	171	170	174	121	121
	T142	193	195	203	205	150	159	152	154	175	175	174	176	121	124
	T49	193	193	187	191	147	154	152	**156**	157	175	170	174	121	121
	T52	193	193	187	189	**144**	149	154	154	175	175	174	176	121	124
	T53	189	193	189	191	**144**	150	154	154	167	175	174	174	121	121
	T55	191	193	188	191	152	152	154	159	157	**173**	176	176	121	128
	T56	193	193	188	193	**144**	152	154	**156**	168	171	168	174	121	121
	T61	193	193	189	193	148	152	154	152	171	171	170	176	121	124
	T63	193	193	185	193	150	152	154	159	171	175	170	180	121	124
	T67	191	193	191	203	150	152	150	152	171	175	168	172	121	124
	T70	189	197	185	191	150	152	152	154	167	169	174	174	121	121
	T83	193	195	189	191	152	154	154	154	157	171	168	170	121	129

Bold are genotypes of the haplotype segregating with *BRCA1* c.815_824dup10 mutation.

We then estimated the age of the pathogenic variant in number of generations, using the following formula G = logδ/log (1 − θ) as described in the methodology. The pathogenic variant is supposed to appear in Senegal 55.5 generations ago, ~1400 years.

## Discussion

Breast cancer in sub-Saharan Africa has a clinical epidemiology characterized by an early age at diagnosis (<50 years), aggressive tumors with poor prognosis.^2,24,25^ This particular epidemiology has been observed in our study population. The average age of diagnosis was 39.5 years and is the lowest reported in sub-Saharan Africa: 45 years in Senegal and Nigeria,^25^ 46 years in Mali,^26^ and 49 years in Ghana.^27^ In Canada, the US, and Australia there has been an average age at diagnosis of about 45 years for sporadic breast cancer and 39.9 years for inherited breast cancer.^28^ A study conducted in Senegal in 2017 has reported a mean age at diagnosis of 47.5 years in women with sporadic breast cancer.^29^ The average age observed in this study is then concordant with that reported in inherited breast cancer over the world.

Tumors characteristics showed a late stage diagnosis with aggressive tumors of bad prognosis mainly grade SBR II and III. This has also been reported in sporadic breast cancer in sub-Saharan Africa.^30–37^ This late stage diagnosis could be linked to the lack of awareness programs for breast cancer symptomatology and diagnosis, and the high cost of breast cancer therapy, most patients first resorted to traditional medicine.

Despite the need expressed by gynecologists and oncologists, molecular phenotyping of tumors is not available in most sub-Saharan African countries. Available data came from abroad at expensive costs inaccessible to most patients. For the few laboratories that have local facilities, the poor quality of biopsies, the inadequacy of fixation time, the lack of equipment and specialists, are pitfalls for achieving these analyzes.^2^ Our results showed that the majority of tumors were triple negative (42.8%) or Her2 enriched (28.5%), all considered as poor prognostic tumors. Added to this, is the young age at diagnosis with 40.7% of our population under 35 years of age. It has been reported that breast cancer diagnosed in women under 40 years with triple negative or Her2-enriched tumors, is of poor prognosis and high metastasis incidence.^38–41^ It therefore appears that breast cancer in young women could constitute a biologically different entity. Germline mutations of *BRCA1* and *BRCA2* genes, and a familial aggregation are frequently observed in this case.^42,43^ Therefore, a *BRCA* gene mutation will be suspected, when an index case has a young age at diagnosis, and/or a family history of breast or ovarian cancer and a poor prognosis tumor. Patients in our study were relatively young (mean age 39.5 years) and all had at least one affected relative, while 14.8% had associated ovarian cancer. These characteristics are in favor of *BRCA* genes inherited mutations and thus a genetic predisposition.

Hereditary breast cancer accounts for 5–10% of breast cancers in women.^1^ Molecular genetics have led to a better understanding of the genetic basis of predisposition to breast/ovarian cancer. Several genes have been involved with two major genes *BRCA1* and *BRCA2* and minor genes *PALB2, P53, PTEN, PALB2, P53, PTEN, CDH1, RAD51, MLH1, MSH2, PMS2, and EPCAM*. Pathogenic variants of these genes play an important role in the genetic predisposition to breast or ovarian cancer. The cumulative risk of breast cancer at age 80 is estimated at 72% for *BRCA1* carriers and 69% for *BRCA2* carriers, while for ovarian cancer it is estimated at 44% for *BRCA1* carriers and 17% for *BRCA2* carriers.^44^

Predisposition to inherited breast cancer has been conducted in European or American populations while in Africa particularly in sub-Saharan Africa, very few studies have been conducted. We report here for the first time in Senegal, a recurrent pathogenic variant of the *BRCA1* gene, c.815_824dup10 involved in the predisposition to hereditary breast cancer. Allelic frequency of the mutation was estimated to 27.7%. This is the highest allelic frequency of this mutation reported in a population. Review from the literature has shown that the variant has been described for the first time in a breast cancer patient from Ivory Coast living in the US.^45^ Later it was also reported in patients from Mexico and the Bahamas,^16^ among African-Americans and Hispanics living in the US,^46^ in Peru,^17^ but also in some European populations. Among African Americans with hereditary breast cancer, it is the most common mutation with a frequency of 16%.^46^ According to CIMBA database, 65 people carrying the variant have been identified worldwide, mostly of African or Hispanic origin. Studies in West Africa, particularly in Nigeria^13^ and Burkina Faso^12^ have not report it in these populations.

When a mutation is identified at a high frequency within a population, exploring for its founding effect becomes important for cancer prevention. Several founder mutations have been reported in different populations. Variants c.66_67delAG and c.5266dupC of *BRCA1* have been used for breast cancer prevention among Jewish women.^13^ In Africa, founder mutations have been reported in Yoruba population from Nigeria (c.303T>G;(p.Tyr101Ter)) in a series of four families,^13^ in Afrikaners from South Africa (c.2641G>T (p.Glu881Ter))^14^ in five families, and in Morocco (c.5309G>T; (p.Gly1770Val)) in five families.^11^ The founding effect of the variant c.815_824dup10 was reported first in five nonrelated families from US^20^ and the mutation was supposed to be of West African origin.

When we screened for its founder effect in Senegal, we identified a haplotype of about 400 kb containing the variant in hereditary breast cancer patients and not in any of the healthy controls. This Senegalese haplotype is shorter compared with the one identified in the US, which spanned 700 kb, as well as the one associated with the Jewish mutation c.66_67delAG.^21,22^ This suggested that the Senegalese haplotype would be older than the African-American haplotype which was estimated to be 200 years old.^20^ Age estimation of the Senegalese haplotype suggested that the variant c.815_824dup10 arose around 1400 years ago. Then we supposed that the variant appeared first in Senegal and was spread throughout the world by population migration, especially by slave trade. Senegal by its geographical position in West Africa was one of the major ports during the trans-Atlantic slave trade toward the European and the US continents.^47^ But it would be of interest to screen for this mutation in other West African countries. Studies conducted in Nigeria and Burkina Faso have not reported it.^12,13^

While the allelic frequency of c.815_824dup10 in our study is high (27.7%), it is necessary to recruit more index cases and more controls in order to estimate the exact allelic frequency of this variant in Senegalese population.

In this study we identified a founder pathogenic variant involved in predisposition to inherited breast cancer in Senegalese women. Screening of the variant in women at risk in Senegal and other West African countries (Mali, Gambia, Ivory Coast, Ghana, and Benin) will be of interest for breast cancer prevention strategies. It will also lead the ground for oncogenetic counselling in Senegal.

## Methods

### Patients

Female index cases with biopsy-proven breast cancer and family history of breast or ovarian cancer, followed up at the Joliot Curie Institute and the Senology department of Aristide le Dantec Hospital in Dakar were recruited. After free written informed consent all participants were interviewed to collect demographic data and medical history. Twenty-seven unrelated female index cases were included in this study. Pedigrees were drawn with Progeny software and 5 ml blood sample was collected from each participant. Characteristics of each index cases and pedigrees are available upon request. From the 27 index cases recruited a first group of 15 was screened for mutations in all exons of the *BRCA1* and *BRCA2* genes. After identification of a recurrent mutation in this first group, the twelve (12) consecutives index patients were screened for this recurrent mutation by PCR genotyping. For index cases with the *BRCA*1 pathogenic variant, healthy relatives were further recruited for genetic testing after written informed consent.

We have also recruited a control population including 90 healthy women without known cancer who came for routine check-up at the Laboratory of Biology of Le Dantec Hospital and 80 women with sporadic breast cancer and without family history from the Joliot Curie Institute. This study was approved by the ethics committee of Cheikh Anta DIOP University under Protocol 014/2014/CER/UCAD. All participants gave their informed written consent before participation in the study.

### Molecular analysis

#### DNA extraction and Sanger sequencing

Genomic DNA was extracted from whole blood with a QiAmp® DNA blood purification kit (Qiagen). *BRCA*1 and *BRCA2* exons were amplified by PCR with specific primers located in intron/exon boundaries. Twenty-eight fragments covering the 22 coding exons of *BRCA1* and 32 fragments covering the 26 coding exons of *BRCA2* gene were amplified.^48^ The large exons 10 and 11 of *BRCA2* were amplified as two and nine fragments, respectively, while exon 11 of *BRCA1* was amplified as seven fragments (Supplementary Table 1). PCRs were carried out with initial denaturation at 95 °C for 10 min followed by 40 cycles of 95 °C for 30 s, 55 °C for 30 s, and 72 °C for 30 s with a GeneAmp® PCR System 9700 (Applied Biosystems) as described previously.^48^

The PCR products were purified with a MinElute 96UF kit and sequenced using a Big Dye terminator V3.1 sequencing kit on a 3730 Genetic Analyzer (Applied Biosystems, Foster City, CA, USA). Both forward and reverse strands were sequenced. Obtained sequences were compared with *BRCA*1 GenBank reference sequence (NM_007294.3) with Alamut Software. This method was used for the first 15 index cases recruited.

#### PCR genotyping of c.815_824dup10 recurrent pathogenic variant of BRCA1

For the following 12 patients and healthy relatives recruited, the recurrent pathogenic variant identified in the first group was genotyped by PCR with primers 5′-TGCTTGTGAATTTTCTGAGACGG-3′ and 5′-TGAGCATGGCAGTTTCTGCT-3′ using standard PCR conditions, followed by a 4% agarose gel migration during 4 h at 100 V. Individuals with the pathogenic variant showed two fragments at 422 and 412 bp (Supplementary Fig. 2).

#### Haplotype analysis

We selected ten index cases with the pathogenic variant and 15 unrelated healthy controls for haplotype analysis. Seven microsatellite markers flanking the *BRCA1* gene located in 2.30 Mb were genotyped by PCR (locus order: cen-D17S1793-D17S1320-D17S855-D17S1323-D17S1325-D17S951-D17S1183-tel) with fluorescent labelled primers (Supplementary Table 2). The PCR products were analysed in automated sequencer ABI Prim 310 using Genscan 3.1.2 software (Applied Biosystems). Allele sizes are given as size of the PCR amplicons containing the microsatellites. Genotyping was performed by Inqaba Biotec^TM^.

#### Age estimation of the founder mutation

The age of c.815_824dup10 pathogenic variant in generations (G) was calculated using the following equation: *G* = logδ/log (1 − θ). Linkage disequilibrium (*δ*; delta) between the mutation and each of the closest recombinant microsatellite markers D17S855 and D17S1325 was calculated as *δ* = (Pd − Pn)/(1 − Pn), with Pd being the frequency of the ancestral microsatellite marker allele among the chromosomes carrying the mutated *BRCA*1 and Pn being the frequency of that microsatellite allele on chromosomes not carrying the mutation. The symbol θ (Teta) represents the recombination fraction between a marker and the gene.^49^ The genetic distances were inferred from Ensembl database (https://www.ensembl.org/index.html).

### Reporting summary

Further information on experimental design is available in the Nature Research Reporting Summary linked to this paper.

## Supplementary information

Supplementary Information

Reporting Summary

## Data Availability

The datasets used and/or analyzed during the current study are available from the corresponding author on reasonable request. Accession codes for novel BRCA2 mutation. BRCA2 c.5219T>G; p(Leu1740Ter): SCV001132044.1.

## References

[1] Ferlay J (2015). Cancer incidence and mortality worldwide: sources, methods and major patterns in GLOBOCAN 2012. Int J. Cancer.

[2] Brinton LA (2014). Breast cancer in Sub-Saharan Africa: opportunities for prevention. Breast Cancer Res Treat..

[3] Jedy-Agba E, McCormack V, Adebamowo C, Dos-Santos-Silva I (2016). Stage at diagnosis of breast cancer in sub-Saharan Africa: a systematic review and meta-analysis. Lancet Glob. Health.

[4] Rebbeck TR (2016). Inheritance of deleterious mutations at both BRCA1 and BRCA2 in an international sample of 32,295 women. Breast Cancer Res..

[5] Antoniou AC (2008). The BOADICEA model of genetic susceptibility to breast and ovarian cancers: updates and extensions. Br. J. Cancer.

[6] Chen S, Parmigiani G (2007). Meta-analysis of BRCA1 and BRCA2 penetrance. J. Clin. Oncol..

[7] Mavaddat N (2013). Cancer risks for BRCA1 and BRCA2 mutation carriers: results from prospective analysis of EMBRACE. J. Natl Cancer Inst..

[8] Struewing JP (1997). The risk of cancer associated with specific mutations of BRCA1 and BRCA2 among Ashkenazi Jews. N. Engl. J. Med..

[9] Tobias DH (2000). Founder BRCA 1 and 2 mutations among a consecutive series of Ashkenazi Jewish ovarian cancer patients. Gynecol. Oncol..

[10] Ibrahim SS, Hafez EE, Hashishe MM (2010). Presymptomatic breast cancer in Egypt: role of BRCA1 and BRCA2 tumor suppressor genes mutations detection. J. Exp. Clin. Cancer Res..

[11] Quiles F (2016). Identification of a founder BRCA1 mutation in the Moroccan population. Clin. Genet..

[12] Zoure AA (2018). BRCA1 c.68_69delAG (exon2), c.181T>G (exon5), c.798_799delTT and 943ins10 (exon11) mutations in Burkina Faso. J. Public Health Afr..

[13] Zhang B (2009). Evidence for an ancient BRCA1 mutation in breast cancer patients of Yoruban ancestry. Fam. Cancer.

[14] Reeves MD (2004). BRCA1 mutations in South African breast and/or ovarian cancer families: evidence of a novel founder mutation in Afrikaner families. Int J. Cancer.

[15] Olopade OI (2003). Breast cancer genetics in African Americans. Cancer.

[16] Akbari MR (2014). The spectrum of BRCA1 and BRCA2 mutations in breast cancer patients in the Bahamas. Clin. Genet..

[17] Dutil J (2015). The spectrum of BRCA1 and BRCA2 alleles in Latin America and the Caribbean: a clinical perspective. Breast Cancer Res Treat..

[18] Donenberg T (2011). A high prevalence of BRCA1 mutations among breast cancer patients from the Bahamas. Breast Cancer Res Treat..

[19] Abugattas J (2015). Prevalence of BRCA1 and BRCA2 mutations in unselected breast cancer patients from Peru. Clin. Genet..

[20] Mefford HC (1999). Evidence for a BRCA1 founder mutation in families of West African ancestry. Am. J. Hum. Genet..

[21] Neuhausen SL (1996). Haplotype and phenotype analysis of six recurrent BRCA1 mutations in 61 families: results of an international study. Am. J. Hum. Genet..

[22] Friedman LS (1995). Novel inherited mutations and variable expressivity of BRCA1 alleles, including the founder mutation 185delAG in Ashkenazi Jewish families. Am. J. Hum. Genet..

[23] Diop JPD (2019). Novel BRCA2 pathogenic variant c.5219 T > G; p.(Leu1740Ter) in a consanguineous Senegalese family with hereditary breast cancer. BMC Med Genet..

[24] Kantelhardt EJ (2015). A review on breast cancer care in Africa. Breast Care (Basel).

[25] Huo D (2009). Population differences in breast cancer: survey in indigenous African women reveals over-representation of triple-negative breast cancer. J. Clin. Oncol..

[26] Ly M (2012). High incidence of triple-negative tumors in sub-saharan Africa: a prospective study of breast cancer characteristics and risk factors in Malian women seen in a Bamako university hospital. Oncology.

[27] Ohene-Yeboah M, Adjei E (2012). Breast cancer in Kumasi, Ghana. Ghana Med. J..

[28] Goodwin PJ (2012). Breast cancer prognosis in BRCA1 and BRCA2 mutation carriers: an International Prospective Breast Cancer Family Registry population-based cohort study. J. Clin. Oncol..

[29] Reeves HL (2004). Kruppel-like factor 6 (KLF6) is a tumor-suppressor gene frequently inactivated in colorectal cancer. Gastroenterology.

[30] Dickens C (2014). Stage at breast cancer diagnosis and distance from diagnostic hospital in a periurban setting: a South African public hospital case series of over 1,000 women. Int J. Cancer.

[31] Seedhom AE, Kamal NN (2011). Factors affecting survival of women diagnosed with breast cancer in El-Minia Governorate, Egypt. Int J. Prev. Med..

[32] Stapleton JM (2011). Patient-mediated factors predicting early- and late-stage presentation of breast cancer in Egypt. Psychooncology.

[33] Zeeneldin AA, Ramadan M, Gaber AA, Taha FM (2013). Clinico-pathological features of breast carcinoma in elderly Egyptian patients: a comparison with the non-elderly using population-based data. J. Egypt Natl Canc Inst..

[34] Ibrahim NA, Oludara MA (2012). Socio-demographic factors and reasons associated with delay in breast cancer presentation: a study in Nigerian women. Breast.

[35] Tesfamariam A, Gebremichael A, Mufunda J (2013). Breast cancer clinicopathological presentation, gravity and challenges in Eritrea, East Africa: management practice in a resource-poor setting. S Afr. Med. J..

[36] Ismaili N, Elyaakoubi H, Bensouda Y, Errihani H (2014). Demographic, clinical, pathological, molecular, treatment characteristics and outcomes of nonmetastatic inflammatory breast cancer in Morocco: 2007 and 2008. Exp. Hematol. Oncol..

[37] Kemfang Ngowa JD (2011). Breast cancer profile in a group of patients followed up at the radiation therapy unit of the yaounde general hospital, Cameroon. Obstet. Gynecol. Int..

[38] Shannon C, Smith IE (2003). Breast cancer in adolescents and young women. Eur. J. Cancer.

[39] Rapiti E (2005). Survival of young and older breast cancer patients in Geneva from 1990 to 2001. Eur. J. Cancer.

[40] Mallol N, Desandes E, Lesur-Schwander A, Guillemin F (2006). Disease-specific and event-free survival in breast cancer patients: a hospital-based study between 1990 and 2001. Rev. Epidemiol. Sante Publique.

[41] Zhou P, Gautam S, Recht A (2007). Factors affecting outcome for young women with early stage invasive breast cancer treated with breast-conserving therapy. Breast Cancer Res. Treat..

[42] Eisinger F (2004). Identification and management of hereditary predisposition to cancer of the breast and the ovary (update 2004). Bull. Cancer.

[43] Antoniou A (2003). Average risks of breast and ovarian cancer associated with BRCA1 or BRCA2 mutations detected in case Series unselected for family history: a combined analysis of 22 studies. Am. J. Hum. Genet..

[44] Kuchenbaecker KB (2017). Risks of breast, ovarian, and contralateral breast cancer for BRCA1 and BRCA2 mutation carriers. JAMA.

[45] Stoppa-Lyonnet D (1997). BRCA1 sequence variations in 160 individuals referred to a breast/ovarian family cancer clinic. Institut Curie Breast Cancer Group. Am. J. Hum. Genet..

[46] Rebbeck TR (2018). Mutational spectrum in a worldwide study of 29,700 families with BRCA1 or BRCA2 mutations. Hum. Mutat..

[47] Beckles, H. M. *VOYAGES D’ESCLAVES: La Traite Transatlantique des Africains Réduits en Esclavage*. (United Nations EducationaI, Scientifïc and Cultural Organization, 2002).

[48] Noguchi T, Bourdon V, Sobol H (2014). About sequence quality: impact on clinical applications. Genet Test. Mol. Biomark..

[49] Risch N (1995). Genetic analysis of idiopathic torsion dystonia in Ashkenazi Jews and their recent descent from a small founder population. Nat. Genet..

