# Supplementary material for: Evidence for an ancient BRCA1 pathogenic variant in inherited breast cancer patients from Senegal
Source: NPJ Genom Med. 2020 Jan 31;5:8. doi: 10.1038/s41525-020-0114-7 (PMC6994613; doi:10.1038/s41525-020-0114-7)
Supplement: Supplementary file 1 — Supplementary Information [file 41525_2020_114_MOESM1_ESM.pdf]

Supplementary Table I: Primers used for BRCA1 coding exons PCR amplification

Exon	Primers		Fragment Length (bp)
	Primer F	Primer R	
2	TGTAAAACGACGGCCAGTGGACGTTGTCATTAGTTCTTTGG	CAGGAAACAGCTATGACCTCTTTTCTTCCCTAGTATGTAAGGTCA	350
3	TGTAAAACGACGGCCAGTTCTCAGTTCCTGACACAGCAG	CAGGAAACAGCTATGACCAAGTTAGGTGTTTCCTGGGTTATG	315
5	TGTAAAACGACGGCCAGTTCATGGCTATTTGCCTTTTG	CAGGAAACAGCTATGACCTCTACTTTTTCCTACTGTGG	232
6	TGTAAAACGACGGCCAGTGGTTTTCTACTGTTGCTGCATCT	CAGGAAACAGCTATGACCCACCACGTCATAGAAAGTAATTGT	312
7	TGTAAAACGACGGCCAGTCACAACAAAGAGCATACATAGGG	CAGGAAACAGCTATGACCCCCAGCTACTAAGGGGGCTA	370
8	TGTAAAACGACGGCCAGTTGTTAGCTGACTGATGATGGT	CAGGAAACAGCTATGACCCACTTCCCAAAGCTGCCTAC	315
9	TGTAAAACGACGGCCAGTCCTGCCACAGTAGATGCTCA	CAGGAAACAGCTATGACCTGCACATACATCCCTGAACC	312
10	TGTAAAACGACGGCCAGTTGGTCAGCTTTCTGTAATCG	CAGGAAACAGCTATGACCGTATCTACCCACTCTCTTTTCAG	259
11T1	TGTAAAACGACGGCCAGTATATAGCCAGTTGGTTGATTT	CAGGAAACAGCTATGACCCATCACTTCTGGAAAACCACTC	610
11T2	TGTAAAACGACGGCCAGTCCTAGAGATACTGAAGATGTTC	CAGGAAACAGCTATGACCGAATAGAATCACCTTTTGTTT	670
11T3	TGTAAAACGACGGCCAGTCAAACGGAGCAGAATGGTC	CAGGAAACAGCTATGACCGACACTTTAACTGTTTCTAG	621
11T4	TGTAAAACGACGGCCAGTAAGTGTTCAAATACCAGTGAAC	CAGGAAACAGCTATGACCATTAGACTCATTCTTTCCTTG	634
11T5	TGTAAAACGACGGCCAGTCACTCTGGGTCCTTAAAGAAAC	CAGGAAACAGCTATGACCCCTAATCTAAGCATAGCATTC	605
11T6	TGTAAAACGACGGCCAGTGAAATAGGTTCCAGTGATG	CAGGAAACAGCTATGACCCAATGATAATAAATTCTCCTC	620
11T7	TGTAAAACGACGGCCAGTTCTCAGTCTACTAGGCATAGC	CAGGAAACAGCTATGACCCTTGGAAATTTGTAAAATGTGC	494
12	TGTAAAACGACGGCCAGTGTCCTGCCAATGAGAAGAAA	CAGGAAACAGCTATGACCTGTCAGCAAACCTAAGAATGT	280
13	TGTAAAACGACGGCCAGTAATGGAAAGCTTCTCAAAGTA	CAGGAAACAGCTATGACCATGTTGGAGCTAGGTCCTTAC	336
14	TGTAAAACGACGGCCAGTTGTGTATCATAGATTGATGCTTTTGA	CAGGAAACAGCTATGACCAAACAAAAGAAGTATCCTAGAGCAA	394
15	TGTAAAACGACGGCCAGTCATGAGCTGTTTCATTTATGCTTT	CAGGAAACAGCTATGACCGAGCTATTTTTCTAAAGTGGGCTTA	451
16	TGTAAAACGACGGCCAGTAATTCTTAACAGAGACCAGAAC	CAGGAAACAGCTATGACCAAAACTCTTTCCAGAATGTTGT	459
17	TGTAAAACGACGGCCAGTAATAGTTCCAGGACACGTG	CAGGAAACAGCTATGACCACCATGCTGGCCAGGATG	386
18	TGTAAAACGACGGCCAGTAGGCTCTTTAGCTTCTTAGGA	CAGGAAACAGCTATGACCAAGACCTTTTGGTAACTCAGA	290
19	TGTAAAACGACGGCCAGTCTGTCATTCTTCCTGTGCTC	CAGGAAACAGCTATGACCCATTGTTAAGGAAAGTGGTGC	262
20	TGTAAAACGACGGCCAGTTGACGTGTCTGCTCCACTTC	CAGGAAACAGCTATGACCCCTGTGTGAAAGTATCTAGCACTG	310
21	TGTAAAACGACGGCCAGTCAGGTGGTGAACAGAAGAAAAA	CAGGAAACAGCTATGACCGAACATTTCAGCAATCTGAGG	308
22	TGTAAAACGACGGCCAGTTCCCATTGAGAGGTCTTGCT	CAGGAAACAGCTATGACCGAACATTTCAGCAATCTGAGG	308
23	TGTAAAACGACGGCCAGTCAGAGCAAGACCCTGTCTC	CAGGAAACAGCTATGACCACTGTGCTACTCAAGCACCA	312
24	TGTAAAACGACGGCCAGTCCTGGAGTCGATTGATTAGAGC	CAGGAAACAGCTATGACCGAAGGACTGAAGAGTGAGAGGAG	404

Supplementary Table 2: Fluorescent labelled primers used for microsatellite markers genotyping

Microsatellite marker	Primer F	Primer R	Size(bp)	DYE
D17S1183	TTAGCTGTGACCATATTGTATTG	CAATTTAATATGTAGCTAGTGGCT	165	6-FAM
D17S1320	ACTTTCCAGAAAATCTCTGCTC	CCACGTCTTTTCTGTGTTCC	180	HEX
D17S1323	TAGGAGATGGATTATTGGTG	AAGCAACTTTGCAATGAGTG	155	6-FAM
D17S1325	AAAGGTGGCAATTCACAGTTG	GTGATAAAACTCAGTGGTACTC	155	HEX
D17S1793	CCAGCCCAAGGTTTACATC	CACCAGTTCCCTGCGAG	163-169	6-FAM
D17S855	GGATGGCCTTTTAGAAAGTGG	ACACAGACTTGTCCTACTGCC	143	CY3, 5
D17S951	GGCCTCCCAAACTGCTT	TCTACCCCGATGAGCCA	170-188	CY3, 5

SM

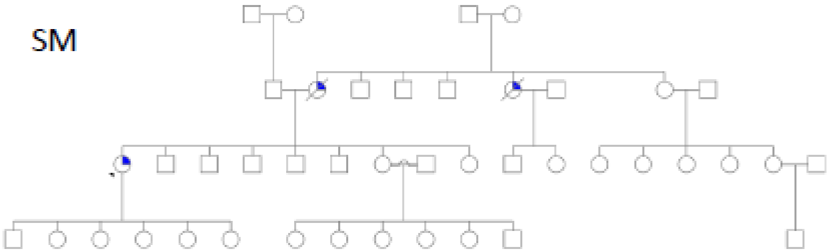

SN

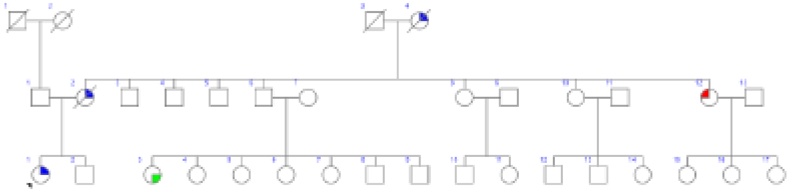

OG

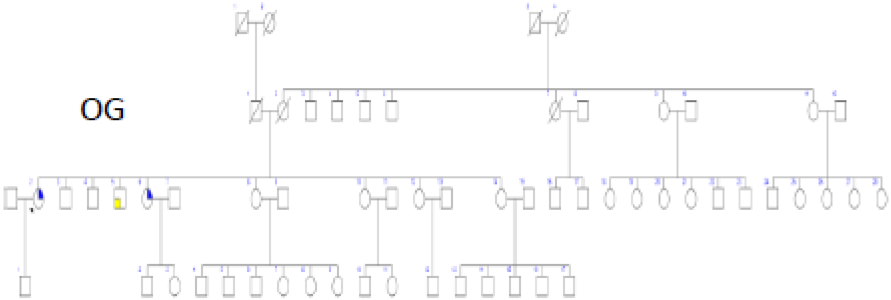

WM

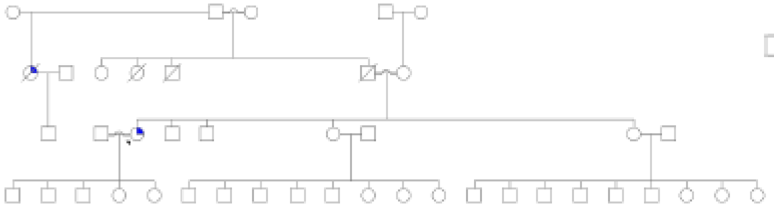

NAD

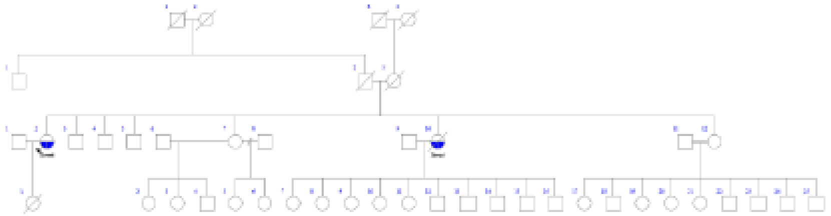

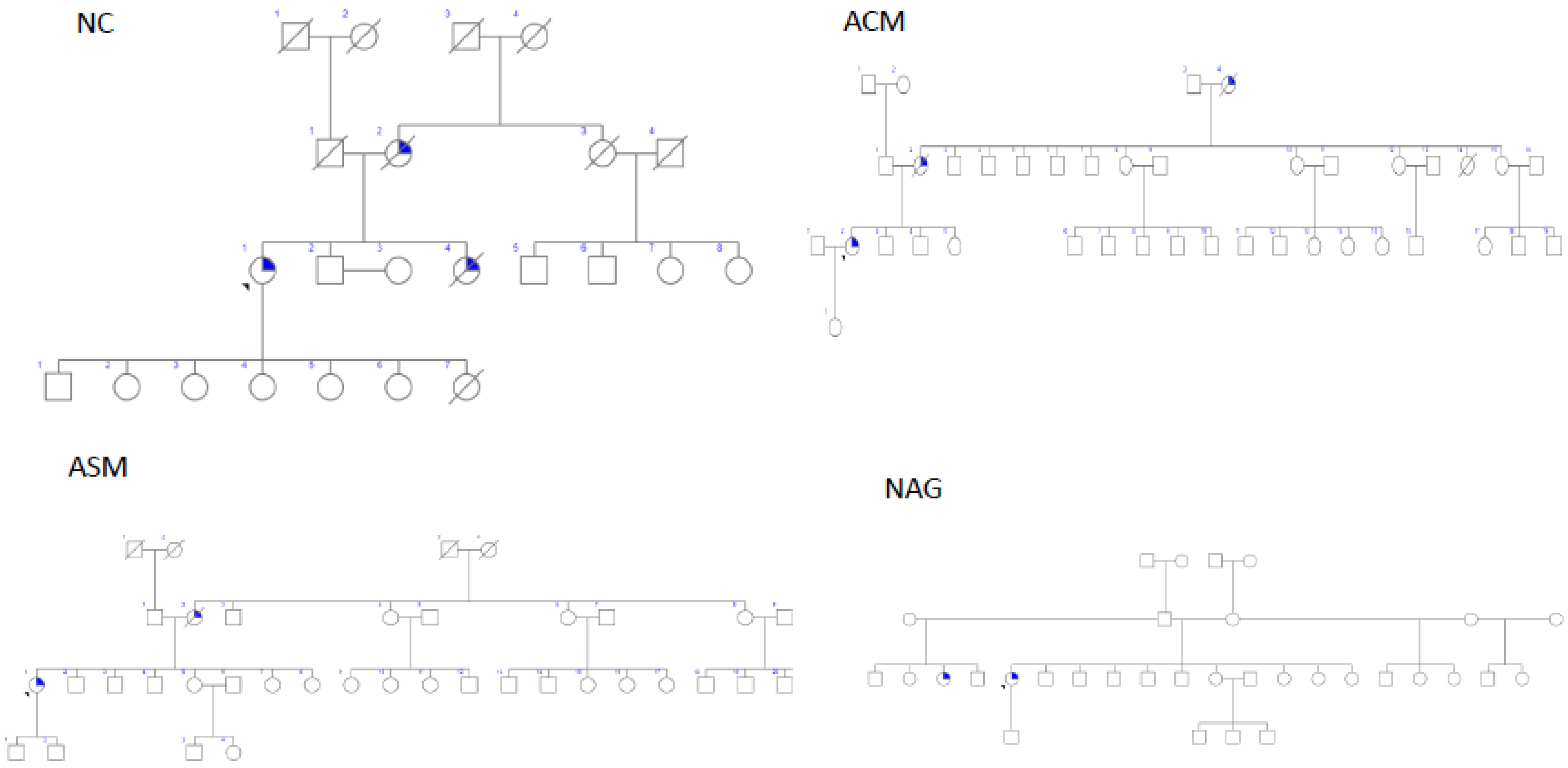

Supplementary Figure 1: Pedigrees of the 9 other *BRCA1* mutation carriers identified by PCR genotyping.

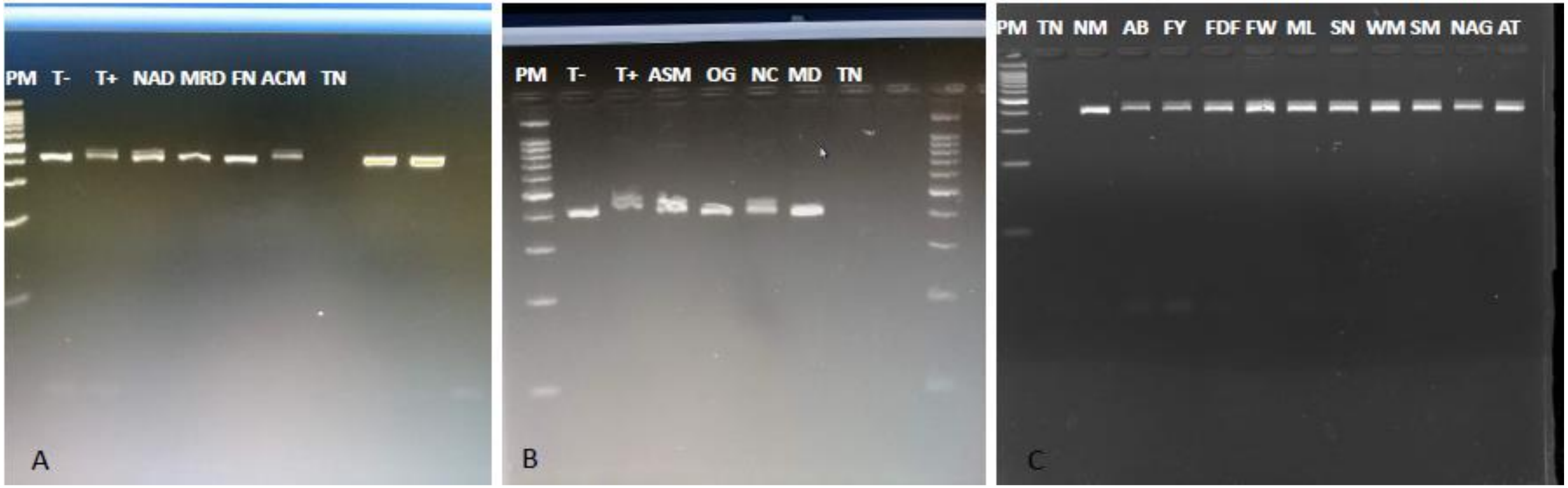

Supplementary Figure 2: Uncropped images of 4% agarose gel electrophoresis of PCR products from the 15 index cases carrying the pathogenic variant

(Each gel derived from a single experiment)

A: NAD-ACM from the second group of 12 recruited index cases

B: ASM-OG-NC from the second group of 12 recruited index cases

C: AB-FY-FGF-FW-ML-AT from the first group of 15 recruited index cases and SN-WM-SM-NAG from the second group of 12 recruited index cases

PM: Molecular weight marker; **TN**: DNA negative control; **T-**: mutation non-carrier control; **T+**: Mutation carrier control
